# Supplementary material for: Segmentation Uncertainty Estimation as a Sanity Check for Image Biomarker Studies
Source: Cancers (Basel). 2022 Mar 2;14(5):1288. doi: 10.3390/cancers14051288 (PMC8909427; doi:10.3390/cancers14051288)
Supplement: Supplementary file 1 [file cancers-14-01288-s001.zip › cancers-1578058-supplementary.pdf]

Table S1. Radiomic features ranked according to their η values in Lung1 set. The top four and the bottom four features were used in the model setup (highlighted in green).

feature	η
original_firstorder_Maximum	0.0
original_gldm_GrayLevelNonUniformity	0.0108
original_glrlm_GrayLevelNonUniformity	0.0115
original_ngtdm_Coarseness	0.0129
original_glszm_LargeAreaHighGrayLevelEmphasis	0.0218
original_glszm_ZoneVariance	0.0277
original_glszm_LargeAreaEmphasis	0.0278
original_firstorder_90Percentile	0.0342
original_glszm_LargeAreaLowGrayLevelEmphasis	0.0356
original_shape_Maximum3DDiameter	0.0394
original_gldm_DependenceVariance	0.0415
original_shape_Maximum2DDiameterColumn	0.0454
original_shape_Maximum2DDiameterRow	0.0455
original_shape_MeshVolume	0.0455
original_shape_VoxelVolume	0.0461
original_shape_Maximum2DDiameterSlice	0.0472
original_firstorder_Median	0.0524
original_ngtdm_Busyness	0.0535
original_glrlm_RunVariance	0.0544
original_shape_SurfaceArea	0.057
original_gldm_LargeDependenceHighGrayLevelEmphasis	0.0628
original_gldm_DependenceNonUniformityNormalized	0.063
original_gldm_LargeDependenceEmphasis	0.0658
original_glrlm_LongRunEmphasis	0.0663
original_glrlm_LongRunHighGrayLevelEmphasis	0.0717
original_shape_SurfaceVolumeRatio	0.0719
original_glrlm_RunPercentage	0.0758
original_glcm_Idmn	0.0791
original_glcm_Idn	0.0798
original_glcm_MaximumProbability	0.0858
original_gldm_DependenceNonUniformity	0.0879
original_glszm_GrayLevelNonUniformity	0.0904
original_glcm_Idm	0.0906
original_ngtdm_Contrast	0.0911
original_glrlm_RunLengthNonUniformityNormalized	0.0913
original_glcm_Id	0.0917
original_glrlm_ShortRunEmphasis	0.093
original_glcm_JointEnergy	0.0933
original_glrlm_RunLengthNonUniformity	0.0957
original_glszm_ZonePercentage	0.0995
original_gldm_SmallDependenceEmphasis	0.0996
original_glcm_InverseVariance	0.1
original_gldm_SmallDependenceLowGrayLevelEmphasis	0.1063
original_firstorder_Range	0.1132
original_firstorder_Uniformity	0.1135

**original_glcm_DifferenceAverage**	0.1146
**original_gldm_HighGrayLevelEmphasis**	0.1153
**original_firstorder_InterquartileRange**	0.1157
**original_glcm_Autocorrelation**	0.1178
**original_glrlm_HighGrayLevelRunEmphasis**	0.1197
**original_ngtdm_Strength**	0.1203
**original_glcm_DifferenceEntropy**	0.1237
**original_firstorder_Mean**	0.1278
**original_glcm_JointEntropy**	0.1313
**original_glcm_JointAverage**	0.1324
**original_glcm_SumAverage**	0.1324
**original_firstorder_Kurtosis**	0.1329
**original_glcm_Contrast**	0.1348
**original_shape_Sphericity**	0.1411
**original_firstorder_RobustMeanAbsoluteDeviation**	0.1433
**original_firstorder_Entropy**	0.1452
**original_gldm_LowGrayLevelEmphasis**	0.1459
**original_glcm_SumEntropy**	0.1479
**original_glrlm_GrayLevelNonUniformityNormalized**	0.1488
**original_glrlm_ShortRunLowGrayLevelEmphasis**	0.1503
**original_glszm_SizeZoneNonUniformityNormalized**	0.1514
**original_glrlm_ShortRunHighGrayLevelEmphasis**	0.1524
**original_glcm_DifferenceVariance**	0.1526
**original_glrlm_LowGrayLevelRunEmphasis**	0.1559
**original_firstorder_Minimum**	0.1564
**original_glszm_HighGrayLevelZoneEmphasis**	0.1569
**original_glszm_SmallAreaHighGrayLevelEmphasis**	0.1577
**original_gldm_LargeDependenceLowGrayLevelEmphasis**	0.1585
**original_glszm_SmallAreaEmphasis**	0.1593
**original_glszm_SizeZoneNonUniformity**	0.1636
**original_firstorder_Skewness**	0.1647
**original_glcm_Imc1**	0.1658
**original_firstorder_10Percentile**	0.1665
**original_gldm_SmallDependenceHighGrayLevelEmphasis**	0.1678
**original_firstorder_MeanAbsoluteDeviation**	0.1803
**original_firstorder_RootMeanSquared**	0.1829
**original_glcm_SumSquares**	0.1908
**original_glcm_ClusterTendency**	0.1966
**original_gldm_GrayLevelVariance**	0.1982
**original_firstorder_Variance**	0.1984
**original_ngtdm_Complexity**	0.2172
**original_glcm_ClusterProminence**	0.2222
**original_glszm_LowGrayLevelZoneEmphasis**	0.2326
**original_gldm_DependenceEntropy**	0.2385
**original_glcm_ClusterShade**	0.2423
**original_glrlm_GrayLevelVariance**	0.249
**original_glszm_SmallAreaLowGrayLevelEmphasis**	0.255
**original_glrlm_LongRunLowGrayLevelEmphasis**	0.2567
**original_firstorder_TotalEnergy**	0.2572

**original_firstorder_Energy**	0.2572
**original_glszm_ZoneEntropy**	0.2726
**original_glcm_Imc2**	0.2816
**original_glcm_Correlation**	0.2874
**original_glszm_GrayLevelVariance**	0.2944
**original_glrlm_RunEntropy**	0.3066
**original_glcm_MCC**	0.3216
**original_glszm_GrayLevelNonUniformityNormalized**	0.4005
